# Efficacy and safety comparison between axillary lymph node dissection with no axillary surgery in patients with sentinel node-positive breast cancer: a systematic review and meta-analysis

**DOI:** 10.1186/s12893-023-02101-8

**Published:** 2023-07-26

**Authors:** Yu-Jia Fan, Jin-Cheng Li, De-Miao Zhu, Hai-Long Zhu, Yi Zhao, Xin-Bing Zhu, Gang Wu, Ting-ting Bai

**Affiliations:** 1 Thyroid & Breast Surgery Department,, Liaocheng People’s Hospital, Liaocheng, 252000 Shandong Province P. R. China; 2 Department of Breast Surgery, the First Affiliated Hospital of Jinzhou Medical University, Jinzhou, 121001 P. R. China

**Keywords:** Axillary lymph node dissection, Sentinel lymph node biopsy, Breast cancer

## Abstract

**Background:**

This systematic review and meta-analysis aimed to study the evidence on the efficacy and safety of omitting axillary lymph node dissection (ALND) for patients with clinically node-negative but sentinel lymph node (SLN)-positive breast cancer using all the available evidence.

**Methods:**

The Embase, Medline, and Cochrane Library databases were searched through February 25, 2023. Original trials that compared only the sentinel lymph node biopsy (SLNB) with ALND as the control group for patients with clinically node-negative but SLN-positive breast cancer were included. The primary outcomes were axillary recurrence rate, total recurrence rate, disease-free survival (DFS), and overall survival (OS). Meta-analyses were performed to compare the odds ratio (OR) in rates and the hazard ratios (HR) in time-to-event outcomes between both interventions. Based on different study designs, tools in the revised Cochrane risk of bias tool were used for randomized trials and the risk of bias in nonrandomized studies of interventions to assess the risk of bias for each included article. Funnel plots and Egger's test were used for the publication’s bias assessment.

**Results:**

In total, 30 reports from 26 studies were included in the systematic review (9 reports of RCTs, 21 reports of retrospective cohort studies). According to our analysis, omitting ALND in patients with clinically node-negative but SLN-positive breast cancer had a similar axillary recurrence rate (OR = 0.95, 95% confidence interval (CI): 0.76–1.20), DFS (HR = 1.02, 95% CI: 0.89–1.16), and OS (HR = 0.97, 95% CI: 0.92–1.03), but caused a significantly lower incidence of adverse events and benefited in locoregional recurrence rate (OR = 0.76, 95% CI: 0.59–0.97) compared with ALND.

**Conclusion:**

For patients with clinically node-negative but SLN-positive breast cancer (no matter the number of the positive SLN), this review showed that SLNB alone had a similar axillary recurrence rate, DFS, and OS, but caused a significantly lower incidence of adverse events and showed a benefit for the locoregional recurrence compared with ALND. An OS benefit was found in the Macro subset that used SLNB alone versus complete ALND. Therefore, omitting ALND is feasible in this setting.

**Trial registration:**

CRD 42023397963

**Supplementary Information:**

The online version contains supplementary material available at 10.1186/s12893-023-02101-8.

## Background

Axillary lymph node dissection (ALND) has been one of the standard treatments for breast cancer during the twentieth century to prevent the dissemination of breast cancer [1]. However, the management of the axilla in breast cancer has evolved recently, with sentinel lymph node biopsy (SLNB) becoming the standard of care for patients clinically negative for axillary lymph node metastasis [2–4]. For patients with negative sentinel lymph node (SLN), omitting ALND was the consistent choice for most management guidelines [5–7]. In contrast, patients with positive SLN were thought to be at risk of further axillary metastases or decreased overall survival (OS) [8]. The consistent guideline on this was as follows: 1) if positive sentinel lymph node for micrometastasis, no further axillary surgery. Besides, if the patients were 1) no preoperative systemic therapy, 2) tumor size < 5 cm, 3) ≤ 2 positive sentinel lymph nodes, 4) breast-conserving therapy planned, and 5) whole-breast radiation planned [9]. However, ALND has traditionally been advocated for patients with positive SLN [10]. Furthermore, it was reported that ALND may be safely spared in a select cohort of patients [11].

The role of ALND is questioned for disease-free survival (DFS) and OS for selected SLNB positive patients compared with SLNB alone. The Z0011 trial carried out by Giuliano et al., indicated that SLNB without ALND could offer excellent regional control and survival for selected patients with early-stage breast cancer [12–14]. It might be feasible to omit ALND in selected patients with positive SLNB. However, there is no evidence on the optimal management of these patients for patient selection according to the histopathological classification.

The histopathological classification of SLN metastasis included macrometastasis (Macro), micrometastasis (Micro), and isolated tumor cells (ITCs) [15, 16]. Originally, nodal metastasis of > 2 mm in largest diameter was defined as Macro, metastasis between > 0.2 mm but ≤ 2.0 mm was defined as Micro and metastasis of ≤ 0.2 mm was defined as ITCs. The prognostic results refer to the different histopathological classification (Macro versus Micro/ITCs) should be different based on their biological characteristics [17].

Some systematic reviews focused on the efficacy of omitting ALND for selected patients with positive SLN [10, 18–20]. However, few have reported the safety of the procedure. In addition, most focused on studies reported 5 years ago and some meaningful observational studies were excluded. However, no study has tried to explore the difference between different histopathological categories of SLN metastasis. The present systematic review and meta-analysis aimed to study the evidence in the literature on the efficacy and safety of omitting ALND for patients with clinically negative axillary lymph node metastasis but positive SLN using all the available evidence. In addition, the feasibility of patient selection for the histopathological classification of SLN metastasis was studied to provide further useful evidence for decision-making in practice.

## Methods

The PRISMA statement was followed for systematic reviews and meta-analyses to report the present study [21, 22]. An ethics review was waived due to the retrospective and anonymous characteristics of the study.

### Criteria for considering studies for this review

The study inclusion and exclusion criteria included: (1) original comparative research studies in full reports, including retrospective or prospective studies and randomized controlled trials (RCTs). Letters, commentaries, conference abstracts, or reviews were excluded; (2) patients with clinically node-negative breast cancer; (3) patients with SLNB positive early-stage breast cancer. Papers with no information of SLNB were excluded; (4) SLNB without ALND as the experimental group; (5) ALND as the control group; and (6) data on any of the following outcomes: OS, progressive-free survival, local recurrence, and adverse events (e.g., lymphedema, sensory neuropathy, motor neuropathy, and infection). In addition, papers without information based on the outcomes or the data that could not be analyzed were excluded.

### Search strategies and study selection

The electronic databases searched to identify reports of relevant clinical trials included the Cochrane CENTRAL Register of Controlled Trials in the Cochrane Library (searched on 25 February 2023), Ovid MEDLINE (1946 to 05 August 2021), and Ovid Embase (1974 to 25 February 2023). The key search terms included sentinel lymph node biopsy (SLNB), breast neoplasms, and lymph node excision. The details of the search strategies can be found in the Additional file 1. In addition, potentially eligible studies were identified by searching the reference lists of retrieved papers.

After omitting duplicated studies, two independent reviewers performed the title and abstract screening for potentially eligible studies using Endnote version X9. Then, full texts for all potentially eligible studies were retrieved to identify studies that met the inclusion and exclusion criteria. Disagreements between both reviewers were referred to a third party.

### Outcomes

The primary outcomes included axillary recurrence rate, the total recurrence (e.g., axillary recurrence, local recurrence, and distance metastasis) rate, DFS, and OS. The second outcome was adverse events.

### Data collection

Data collection was carried out independently using the pre-test data collection form. Data collection referred to the information of the included studies, which included study design, publication year, country, study design, target population, age of related patients, clinical tumor stages, hormone receptors, nodal metastasis (micro or macro), experimental and control intervention, other interventions, and outcomes.

### Study quality assessment

The RCTs’ quality was assessed using the revised Cochrane risk of bias tool for randomized trials (RoB2) and the quality of nonrandomized studies used the risk of bias in nonrandomized studies of interventions (ROBIN-I) tool [23–25].

### Data analysis

The continuous variables were described with mean and standard deviation (SD), and categorical variables with count with percentage/proportion. For dichotomous data, summary estimates were expressed as odds ratio (OR) with 95% CI (this was carried out for the axillary recurrence rate, total recurrence rate, total survival rate, and adverse events rate). For time-to-event data, the summary estimates were presented as hazard ratio (HR) with 95% CI (this was carried out for time to DFS and time to OS).

For missing data, the study authors were contacted. Then, if the data was insufficient for analysis, descriptive data were presented in the systematic review. Missing data were not used; therefore, studies were excluded that did not have any available data for outcome analysis.

Data analysis was performed using Stata, version 15.0 (Stata Corp. Texas, USA), and Review Manager (Revman) 5.4.1 software. Meta-analysis was performed for outcome measures if ≥ 2 clinically homogenous studies (e.g., studies with similar participants, interventions, and outcomes). Heterogeneity was estimated using the Q-test and I^2^ score. When the *p*-value was < 0.1 (for Q-test) and I^2^ > 50%, the result was considered with heterogeneity, and the random-effects model was used for analysis. Otherwise, a fixed-effects model was applied for analysis [26–28]. A *p*-value < 0.05 was set as the threshold for statistical significance. Subgroup analysis was performed according to the classification of the nodal metastasis (Micro and ITCs versus Macro). Sensitivity analysis was performed according to the methodology quality of the included papers.

Funnel plots generated by Revman 5 confirmed symmetry for the publication bias. If there was an asymmetry of the funnel plot, Egger’s test using Stata version 15 was performed. If the *p-*value < 0.05 of the Egger’s test, which suggested the publication bias existed, this was dealt with using the trim-and-fill method [29].

## Results

### Study selection and characteristics of included studies

**Fig. 1:**
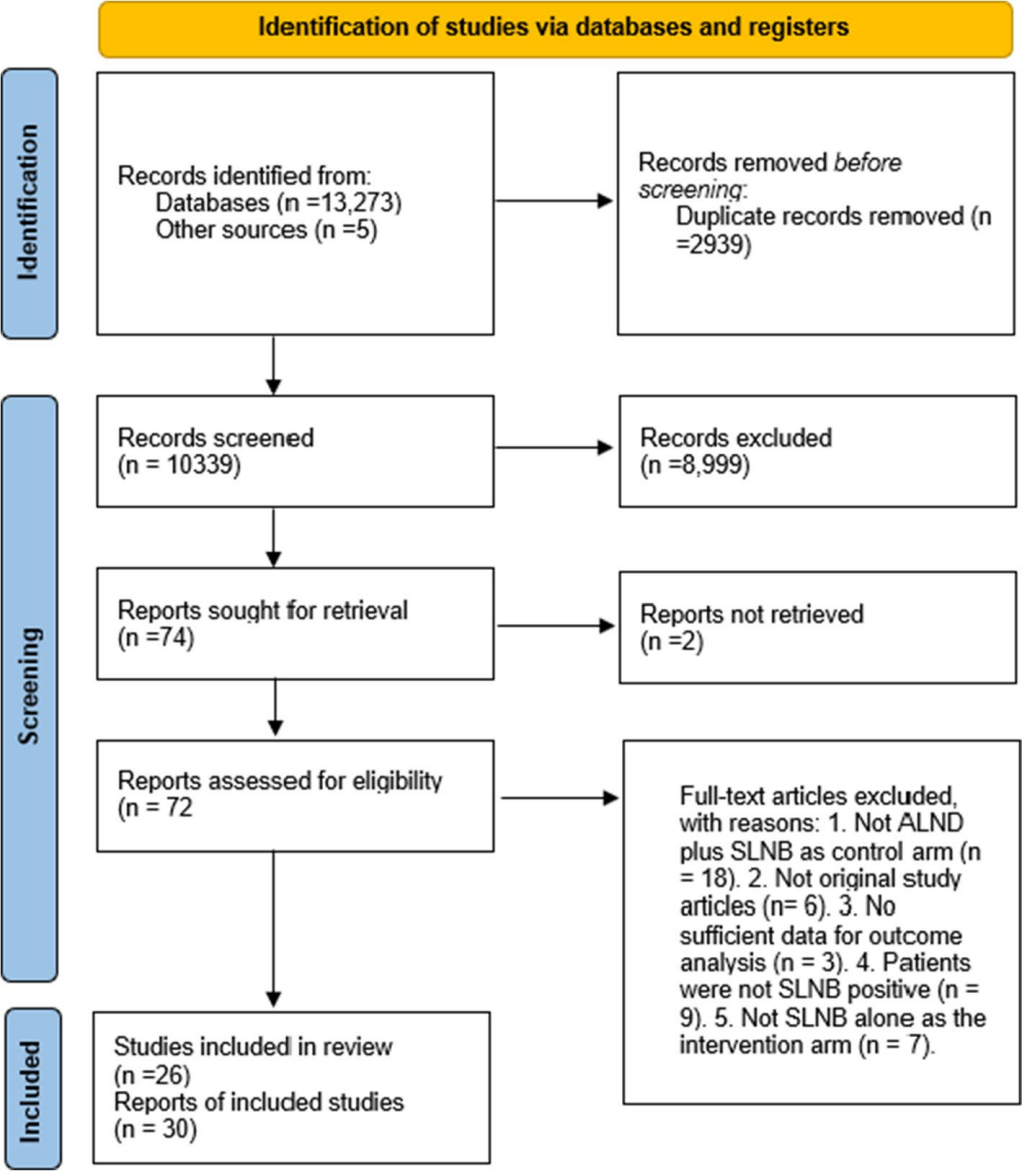
PRISMA flow chart of the procedure for eligible study selection [50]

The flow chart of eligible study selection is shown in Fig. 1. A total of 13,273 studies (1306 studies from Cochrane Library CENTRAL, 5,006 from Medline, 6,961 from Embase, and 5 studies from other sources) were found. After excluding duplications, 10,339 studies were used for the title and abstract screening. Then, 72 papers were retrieved for full-text review. Finally, 30 reports for 26 studies were included in the systematic research and meta-analysis according to the inclusion and exclusion criteria [12, 13, 30–57].

In total, 191,329 patients were involved in the included 26 studies with a sample size from 81 to 26,986 patients. The number of the RCTs and retrospective studies was 5 and 21, respectively. The papers were published between 2007 and 2022. However, not all studies supplied the information of the number of the positive SLN and the previous systematic therapy. The details of the included 26 studies reported in 30 papers are listed in Table 1.

**Table 1:** The characteristics of the included 20 studies reported by 24 papers

**Author publication year**	**Study design**	**Lymph bode category**	**Sample size (n), total (SLNB group/ALND group)**	**Positive SLN, n (SLNB group/ALND group)**	**Age (years), total (SLNB group/ALND group)**	**Tumor size, total (SLNB group/ALND group)**	**Axillary intervention**	**Median follow-up, months or specified**	**Primary outcome(s)**
**Lucci et al. (2007) ACOSOG Z0011 **[30]	RCT	Not specified	821(417/404)	Range: (1–2/1–2)	Median: not specified for total (54/56)	T_2_: 252 (120/132)	SLNB alone versus SLNB + complete ALND	Not specified	Adverse surgical effects
**Bilimoria et al. (2009) **[31]	Retrospective cohort study	Micro	10,259 (3,674/6,585)	Not specified	Median: 56 (58/56)	Median: 2.0 (1.8/2.1) cm	SLNB alone versus SLNB + complete ALND	63	Axillary recurrence and survival
		Macro	87,055 (16,543/70,512)						
**Degnim et al. (2010) **[32]	Retrospective cohort study	ITC	81 (50/31)	Range: (1–3/1–3)	Mean, not specified for total (50/58)	Number of T_2_ + T_3_: 29 (15/14)	SLNB alone versus SLNB + complete ALND	38	Predicted probability of non-sentinel node metastasis and breast recurrence
**Giuliano et al. (2010) ACOSOG Z0011 **[33]	RCT	Micro	280 (160/120)	Range: (1–2/1–2)	Median: not specified for total (54/56)	Number of T_2_: 251 (124/127)	SLNB alone versus SLNB + complete ALND	6.3 years	Locoregional recurrence
		Macro	418 (219/199)						
		Unknown	115 (66/49)						
**Yi et al. (2010) **[34]	Retrospective cohort study	Micro	6,838 (2,240/4,598)	Range: (1–24/1–54)	Median: not specified for total (59/56)	Number of T_2_ + T_3_: 12,166 (1,430/10,736)	SLNB alone versus SLNB + complete ALND	50	OS and breast cancer-specific mortality
		Macro	20,148 (2,185/17,963)						
**Giuliano et al. (2011) ACOSOG Z0011 **[12]	RCT	Not specified	856 (436/420)	Range: (1–2/1–2)	Median: not specified for total (54/56)	Number of T_2_: 260 (126/134)	SLNB alone versus SLNB + complete ALND	6.3 years	OS
**Gillanders et al. (2012) **[35]	Retrospective cohort study	Micro	78 (31/47)	Not specified	Median: not specified for total (57/51)	Number of T_2_ + T_3_: 102 (19/83)	SLNB alone versus SLNB + complete ALND	73 for SLNB group/ 69 for ALND group	Recurrence and breast cancer-specific mortality
		Macro	198 (39/159)						
**Galimberti et al. (2013) IBCSG 23–01 **[13]	RCT	Micro	931 (467/464)	Range: (1–2/1–2)	Median: not specified for total (54/53)	Number of > 2 cm: 281 (140/141)	SLNB alone versus SLNB + complete ALND	5 years	DFS
**Sola et al. (2013) AATRM 048/13/2000 **[36]	RCT	Micro	233 (121/112)	Not specified	Mean, not specified for total (53.2/ 55.3)	Mean, not specified for total (1.78/1.57) cm	SLNB alone versus SLNB + complete ALND	62	DFS
**Park et al. (2014) **[37]	Retrospective cohort study	Not specified	2545 (197/ 2,348)	94.9% ≤ 3/ 74.4% ≤ 3	Mean, not specified for total (47.9/ 48.7)	Number of T_2_: 101 (67/34)	SLNB alone versus SLNB + complete ALND	42	OS
**Snow et al. (2015) **[38]	Retrospective cohort study	Not specified	318 (60/258)	Range: (1–2/1–10)	Median: 54 (58, 53)	Mean, not specified for total (1.86/ 2.35) cm	SLNB alone versus SLNB + complete ALND	45.5	OS and recurrence
**Tvedskov et al. (2015) **[39]	Retrospective cohort study	Micro	1,673 (136/ 1,537)	Not specified	Mean: 77 (71/ 78)	Number of > 2 cm: 562 (55/507)	SLNB alone versus SLNB + complete ALND	Not specified	Axillary recurrence and OS
		ITC	401 (104/ 297)			Number of > 2 cm: 175 (48/127)			
**Houvenaeghel et al. (2016) **[40]	Retrospective cohort study	Micro	1390 (191/1199)	More than 99% ≤ 2	Median: 56 (not specified)	Number of > 2 cm: 264 (39/225)	SLNB alone versus SLNB + complete ALND	60.4	OS and recurrence-free survival
		ITC	619 (147/472)			Number of > 2 cm: 127 (23/104)			
**Youssef et al. (2016) **[41]	Retrospective cohort study	Micro or ITC	95 (57/38)	Range: (1–2/1–2)	Not specified for median or mean	Number of > 2 cm: 41 (23/18)	SLNB alone versus SLNB + complete ALND	34.2	Locoregional recurrence and lymphedema rate
**Giuliano et al. (2017) ACOSOG Z0011 (Alliance) **[42]	RCT	Not specified	856 (436/420)	Not specified	Median: not specified for total (54/56)	Number of T_2_: 260 (126/134)	SLNB alone versus SLNB + complete ALND	9.3 years	10-year OS
**Galimberti et al. (2018) IBCSG 23–01 **[43]	RCT	Micro	931 (467/464)	Range: (1–2/1–2)	Median: not specified for total (54/53)	Number of > 2 cm: 85 (36/49)	SLNB alone versus SLNB + complete ALND	9.7 years	DFS
**Lee et al. (2018) **[44]	Retrospective cohort study	Not specified	4,442 (1,268/3,174)	Range: (1–2/1–2)	Mean: 49.34 (49.55, 49.26)	Mean: 1.86 (1.81/1.88)	SLNB alone versus SLNB + complete ALND	47.24	Disease-specific survival and OS
**Liu et al. (2018) **[45]	Retrospective cohort study	Micro	5,660 (3,689/1,971)	Range: (1/1)	Not specified for median or mean	1709 (1072/637)	SLNB alone versus SLNB + complete ALND	Not specified	Breast cancer-specific survival
**Arisio et al. (2019) **[46]	Retrospective cohort study	Macro	127 (95/32)	Not specified	Median: Median: not specified for total (57/54)	Number of T_2_ + T_3_: 278 (83/195)	SLNB alone versus SLNB + complete ALND	84.4	OS and relapse-free survival
		Micro	199 (115/84)						
**Jung et al. (2019) **[47]	Retrospective cohort study	Not specified	1,697 (707/990)	Range: (1–2/1–2)	Median: 49 (50/50)	Mean: 2.1 (2.0/2.2)	SLNB alone versus SLNB + complete ALND	50	Disease recurrence
**Kim et al. (2019) **[48]	Retrospective cohort study	Not specified	1,697 (1,539/158)	Range: (1–3/1–3)	Mean: 47.70 (47.59/48.77)	Mean: 2.8 (2.9/2.4)	SLNB alone versus SLNB + complete ALND	93	DFS and OS
**Jung et al. (2020) **[49]	Retrospective cohort study	Not specified	23,138 (16,518/6,620)	Range: (1–2/1–2)	Median: 60 (62/59)	Number of T2: 9,464 (6,429/3,035)	SLNB alone versus SLNB + complete ALND	41	Breast cancer-specific mortality
**Kim et al. (2020) **[50]	Retrospective cohort study	Not specified	883 (179/704)	Range: (1–2/1–2)	Mean: 50.80 (50.78/50.81)	Number of T2: 474 (96/378)	SLNB alone versus SLNB + complete ALND	54	OS
**Sun et al. (2021) **[51]	Retrospective cohort study	Not specified	329 (128/201)	Range: (1–2/1–2)	Median: 53 (56/51)	Median: 2.3 (2.1/2.5) cm	SLNB alone versus SLNB + complete ALND	51	locoregional and distant recurrence and OS
**Sanvido et al. (2021) **[52]	Retrospective cohort study	Mixed with Macro, ITC and Micro	97 (56/41)	Range (1–2/1–24)	Mean: 57.8 (58.3/56.3)	Mean: 1.7 (1.8/2/2) cm	SLNB alone versus SLNB + complete ALND	4.3 years	OS and the locoregional recurrence
**Bartels et al. (2022) EORTC 10981–22023 AMAROS Trial **[53]	RCT	Mixed with Macro, ITC and Micro	1425 (681/744)	Median (2/2)	Median: not specified (55/56)	Median: not specified (18/17) mm	SLNB + ART versus SLNB + complete ALND	10.0 years	Axillary recurrence rate
**Gao et al. (2022) **[54]	Retrospective cohort study	Mixed with Macro, ITC and Micro	1050 (245/805)	Range: (1–2/1–2)	Median: 51 (not specified)	Number of T2: 501 (92/409)	SLNB alone versus SLNB + complete ALND	36	Locoregional recurrence
**Houvenaeghel et al. (2022) **[55]	Retrospective cohort study	Micro	1421 (185/1266)	99.5% of the patients were ≤ 2	Median: not specified (58.5/55.5)	> 20 mm: 260 (28/232)	SLNB alone versus SLNB + complete ALND	54	OS and DFS
**Tinterri et al. (2022) SINODAR-ONE trial **[56]	RCT	Macro	879 (440/439)	Range: (1–2/1–2)	Mean 56.2 (56.2/56.1)	Not specified	SLNB alone versus SLNB + complete ALND	34	OS
**Zhou et al. (2022) **[57]	Retrospective cohort study	Micro	13848 (1965/ 1883)	Not specified	Mean: 57.95 (58.14/56.77)	Mean 23.64 (22.46/ 31.17) mm	SLNB alone versus SLNB + complete ALND	48	OS

*Abbreviation*: *SLNB* sentinel lymph node biopsy, *ALND* axillary lymph node dissection, *ART* axillary radiotherapy, *IQR* interquartile range, *RCT* randomized controlled trial, *OS* overall survival, *ITC* SLN isolated tumor cells, *Micro* SLN micro-metastases, *Macro* SLN macro-metastases, *DFS* disease-free survival

### Methodological quality appraisal

The risk of bias in three RCTs is summarized in Table 2. Three of the studies had issues with bias in randomization. Therefore, for the overall assessment, two studies (EORTC 10981–22,023 AMAROS Trial and ACOSOG Z0011) were defined as low risk of bias, and the other three (SINODAR-ONE trial, IBCSG 23–01, and AATRM 048/13/2000) were defined as having some concerns.

**Table 2:** Summary assessment of the risk of bias for included RCTs using the revised Cochrane risk of bias tool for randomized trials (RoB2)

**Author (publication year)**	**Randomization**	**Deviations from intended intervention**	**Missing outcome data**	**Measurement of the outcome**	**Selection of the reported results**	**Overall**
**Lucci et al. 2007, Giuliano et al. 2010, Giuliano et al. 2011, and Giuliano et al. 2017, ACOSOG Z0011 **[12, 30, 33, 42]						
**Galimberti et al. 2013 and Galimberti et al. 2018, IBCSG 23–01 **[13, 43]						
**Sola et al. 2013, AATRM 048/13/2000 **[36]						
**Bartels et al. (2022)** EORTC 10981–22023 AMAROS Trial [53]						
**Tinterri et al. (2022)** **SINODAR-ONE trial **[56]						

*Notes*:  Low risk of bias,  High risk of bias,  Some concerns

For the 21 retrospective studies, the risk of bias assessment results is listed in Table 3. All 21 studies have a moderate risk of bias in the selection of the reported result as none of those retrospective studies had priori protocol published. However, no evidence showed that they had problems in the domains with bias in the selection of participants into the study, bias in the classification of interventions, or bias in the measurement of outcomes. In addition, about half of the studies were defined as serious risk of bias due to the confounding (11/21, 52.4%) and with no information on missing data (11/21, 52.4%). Therefore, the overall risk of bias was defined as moderate and serious risk of bias in 10 (47.6%) and 11 (52.4%) studies, respectively.

**Table 3:** Summary assessment of the risk of bias for 17 retrospective studies using the ROBIN-I tool

**Author publication year**	**Bias due to confounding**	**Bias in the selection of participants into the study**	**Bias in the classification of interventions**	**Bias due to deviations from intended interventions**	**Bias due to missing data**	**Bias in measurement of outcomes**	**Bias in the selection of the reported result**	**Overall risk of bias**
**Bilimoria et al. (2009) **[31]								Moderate risk of bias
**Degnim et al. (2010) **[32]								Serious risk of bias
**Yi et al. (2010) **[34]								Serious risk of bias
**Gillanders et al. (2012) **[35]								Serious risk of bias
**Park et al. (2014) **[37]								Moderate risk of bias
**Snow et al. (2015) **[38]								Serious risk of bias
**Tvedskov et al. (2015) **[39]								Serious risk of bias
**Houvenaeghel et al. (2016) **[40]								Moderate risk of bias
**Youssef et al. (2016) **[41]								Serious risk of bias
**Lee et al. (2018) **[44]								Serious risk of bias
**Liu et al. (2018) **[45]								Moderate risk of bias
**Arisio et al. (2019) **[46]								Serious risk of bias
**Jung et al. (2019) **[47]								Moderate risk of bias
**Kim et al. (2019) **[48]								Serious risk of bias
**Jung et al. (2020) **[49]								Moderate risk of bias
**Kim et al. (2020) **[50]								Moderate risk of bias
**Sun et al. (2021) **[51]								Serious risk of bias
**Sanvido et al. (2021) **[52]								Serious risk of bias
**Gao et al. (2022) **[54]								Moderate risk of bias
**Houvenaeghel et al. (2022) **[55]								Moderate risk of bias
**Zhou et al. (2022) **[57]								Moderate risk of bias

*Notes*:  low risk of bias,  moderate risk of bias,  serious risk of bias,  for critical risk of bias,  no information

### Axillary recurrence rate

**Fig. 2:**
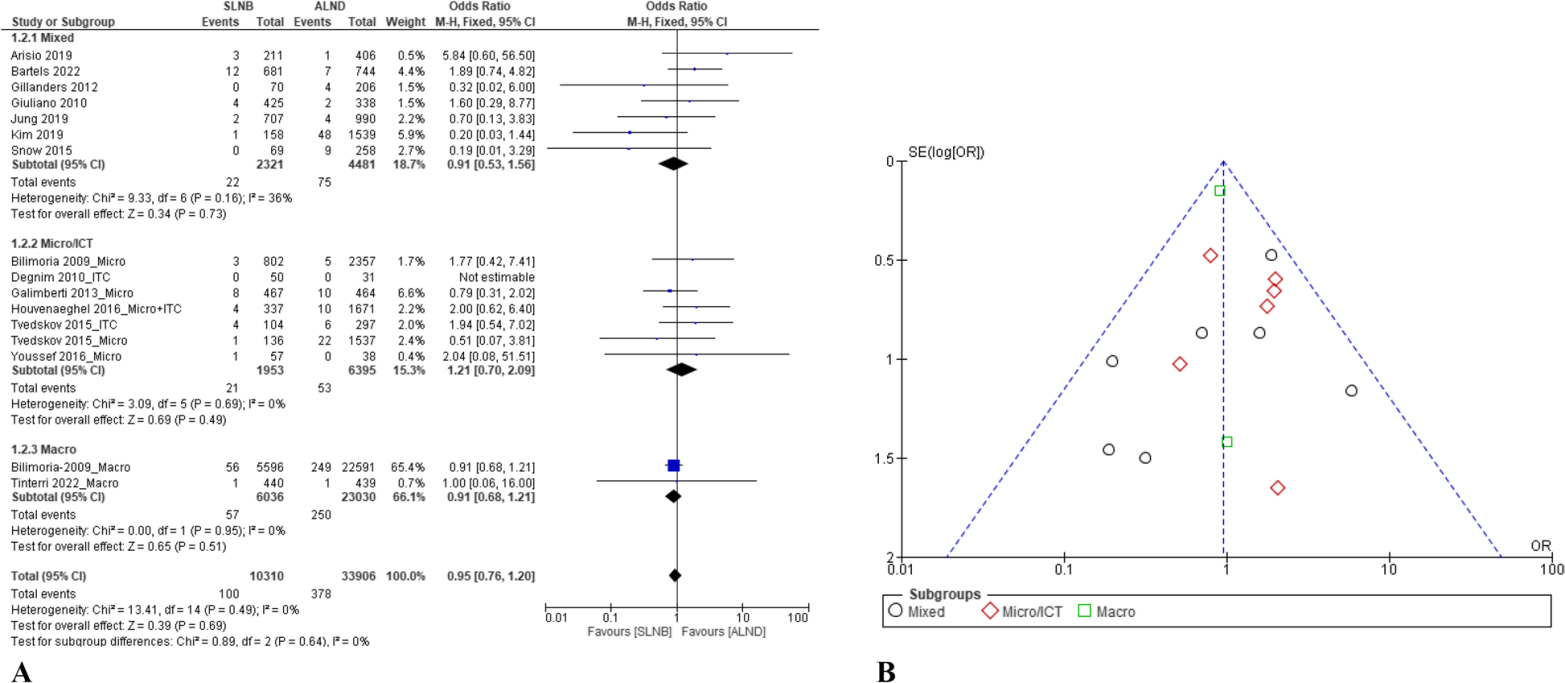
Comparison of axillary recurrence rates between the SLNB alone and complete ALND: **A** forest plots; and **B** funnel plot

Thirteen papers reported data on the comparison of the axillary recurrence rate between both interventions [13, 31–33, 38–41, 46, 48, 49, 53, 56]. The pooling results showed no statistical difference in axillary recurrence rate between both interventions (*p* = 0.69). Compared with the ALND group, the OR of axillary recurrence rate for the SLNB group was 0.95 (95% CI: 0.75–1.20) with a fixed model as *p* = 0.64 (Q-test) and I^2^ = 0%. The subgroup analysis based on the nodal classification showed that although the mean of OR for the Micro/ITC group (1.21, 95% CI: 0.70–2.09) was higher than the Macro group (0.91, 95% CI: 0.68–1.21), there was no statistically significant difference between them (Fig. 2A).

For sensitivity analysis, after deleting the subsets (e.g., the Micro/ITC group and the Macro group), comparing the SLNB group with the ALND group, the OR of the axillary recurrence rate was 0.91 (95% CI: 0.53–1.56). Compared with the original meta-analysis result, there was no statistically significant difference (Fig. 2A). In addition, based on the funnel plot analysis (Fig. 2B), there was no publication bias in the included studies for this outcome.

### Locoregional recurrence rate

**Fig. 3:**
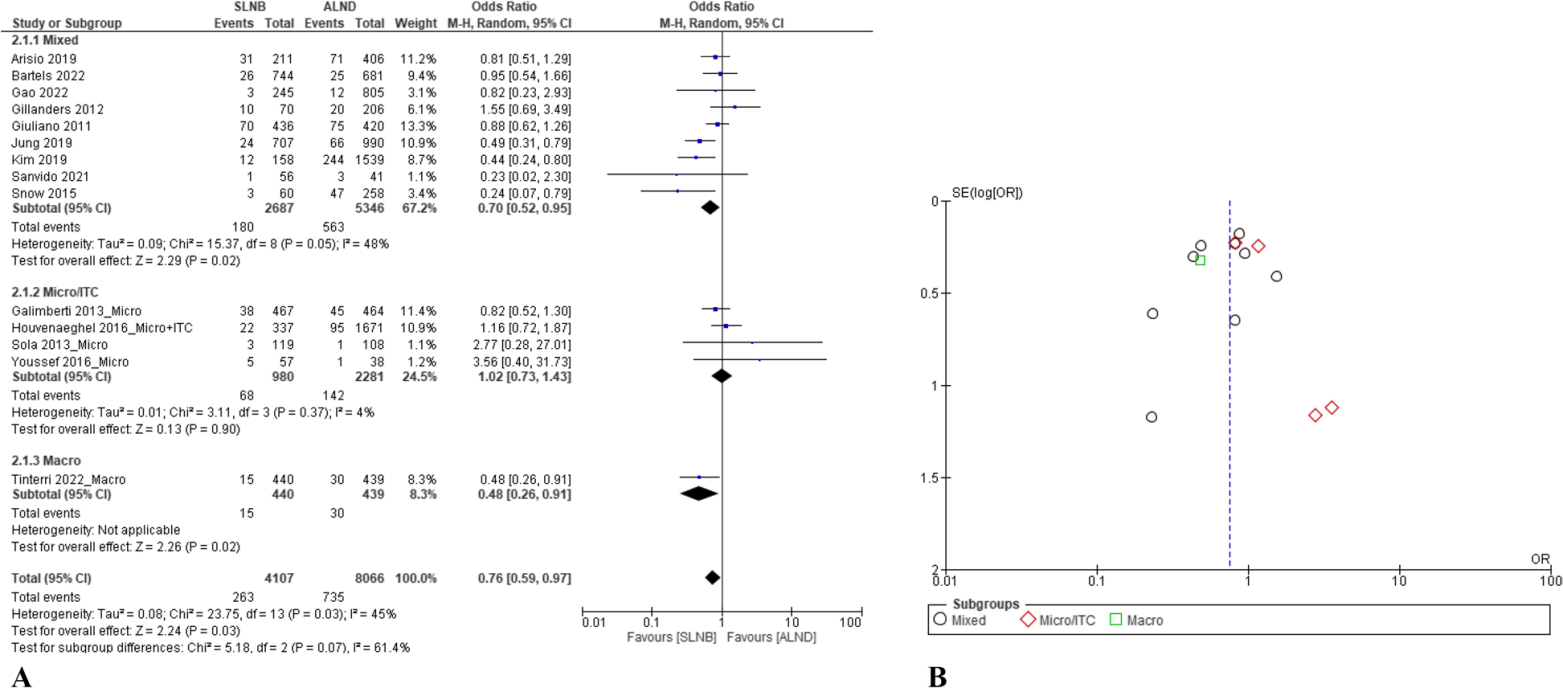
Comparison of total recurrence rates between SLNB alone and complete ALND: **A** forest plots; and **B** funnel plot

Thirteen papers compared the locoregional recurrence rate between both interventions [12, 13, 35, 36, 38, 40, 41, 46, 48, 49, 52, 53, 56]. The pooling results showed benefit for the SLNB group in locoregional recurrence rate (*p* = 0.03). Compared with the ALND group, the OR of locoregional recurrence rate for the SLNB group was 0.76 (95% CI: 0.59–0.97) with the random model as *p* = 0.03 (Q-test) and I^2^ = 45% (Fig. 3A). For patients with the Micro/ITC metastasis, the OR of the locoregional recurrence rate for the SLNB group compared with the ALND group was 1.02 (95% CI: 0.73–1.43). Only one study reported the locoregional recurrence rate for patients with the Macro metastasis, which showed a benefit for the SLNB group (*p* = 0.02) [56].

For sensitivity analysis, after deleting the subset of the Micro/ITC group and Macro group, comparing the SLNB group with the ALND group, the OR of locoregional recurrence rate was 0.70 (95% CI: 0.52–0.95), which was statistically significant (Fig. 3A). In addition, based on the funnel plot analysis (Fig. 3B), there might be publication bias in the included studies for this outcome. However, Egger's test showed that no small-study effect existed for the outcome based on the data (*p* = 0.735). Using the trim-and-fill method, the random effect model showed the result (OR = 0.75, 95% CI: 0.56–0.96, *p* = 0.04) was similar to the original result.

#### DFS

**Fig. 4:**
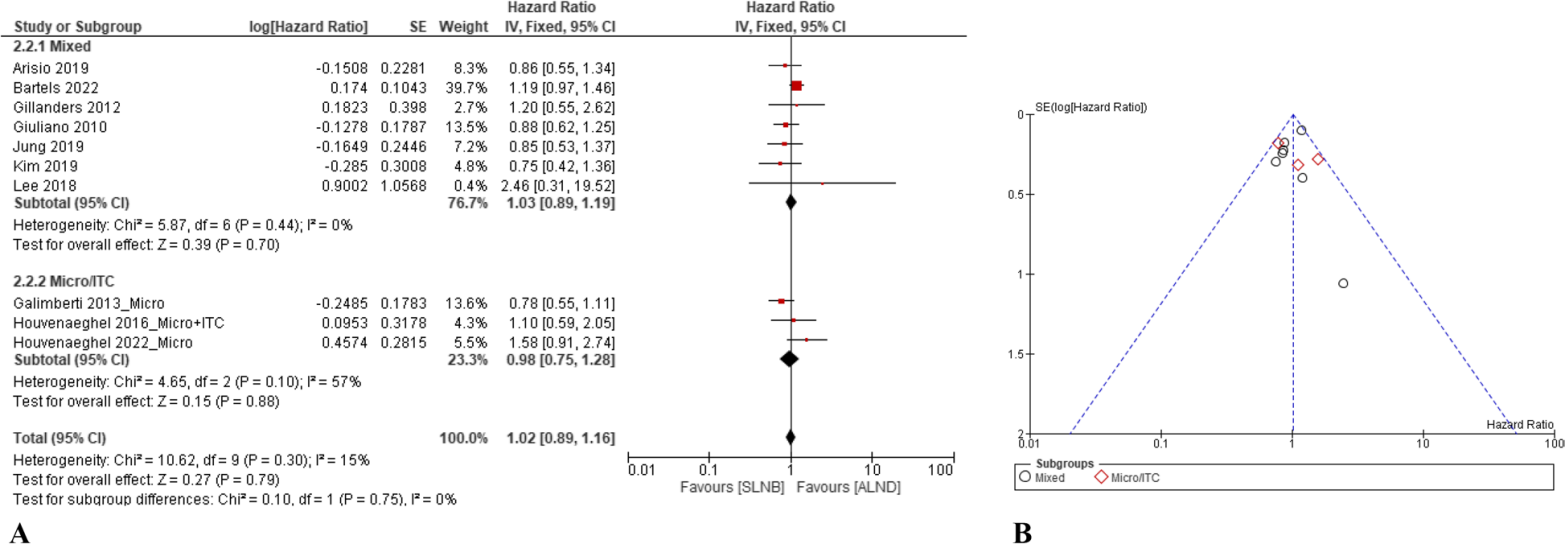
Comparison of DFS between the SLNB alone and complete ALND: **A** forest plots; and **B** funnel plot

Ten papers compared the DFS between patients treated with ALND and SLNB alone[12, 13, 35, 40, 44, 46–48, 53, 55]. A fixed-effect statistical model (Q-test: *p* = 0.75 and I^2^ = 0%) revealed that the DFS was not significantly different between the SLNB group and ALND group (HR = 1.02, 95% CI: 0.89–1.16, *p* = 0.79). Three papers compared the DFS between patients in Micro/ITC treated ALND and SLNB only [13, 40, 55]. No statistical difference for the DFS was found between the two interventions (HR = 0.98, 95% CI: 0.75–1.28, *p* = 0.88) (Fig. 4A).

For sensitivity analysis, after deleting the subset of the Micro/ITC group, comparing the SLNB group with the ALND group, the HR of DFS was 1.03 (95% CI: 0.89–1.19, *p* = 0.70). The funnel plot showed that there was no publication bias that existed (Fig. 4B).

#### OS

**Fig. 5:**
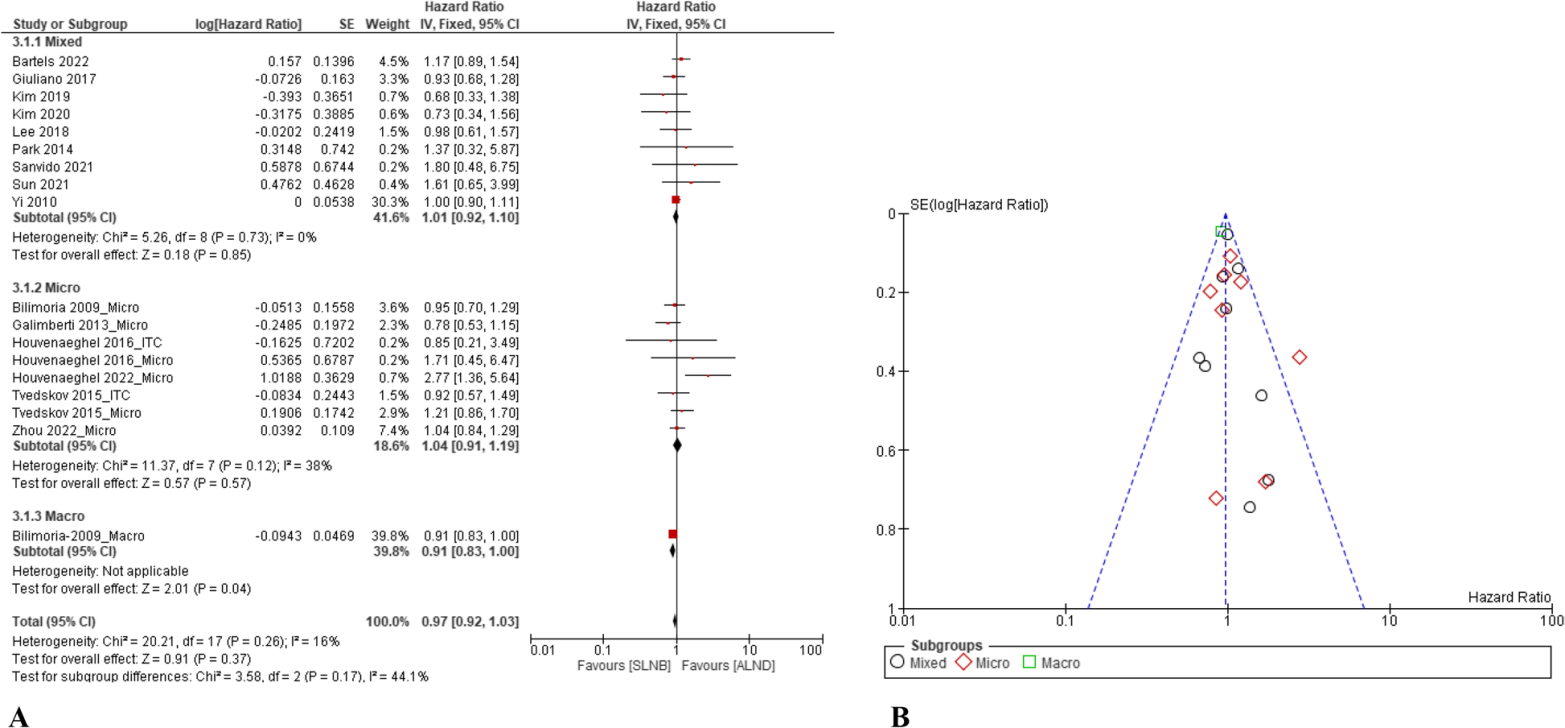
Comparison of OS between the SLNB alone and complete ALND: **A** forest plots; and **B** funnel plot

Fourteen papers compared the OS between patients treated with ALND and SLNB only [13, 31, 34, 37, 39, 40, 42, 44, 48, 50, 51, 53, 55, 57]. A fixed-effect statistical model (Q-test: *p* = 0.26 and I^2^ = 16%) revealed that the OS was not significantly different between the SLNB only and ALND groups (HR = 0.97, 95% CI: 0.92–1.03, *p* = 0.37). Six papers compared the DFS between patients in the Micro/ITC treated ALND and SLNB only groups [13, 31, 39, 40, 55, 57]. No statistical difference for the DFS was found between the two interventions (HR = 1.04, 95% CI: 0.91–1.19, *p* = 0.57) (Fig. 5A). One paper reported a significant increase in the OS that compared SLNB only with the ALND group in patients with Macro (HR = 0.91, 95% CI: 0.83–1.00, *p* = 0.04) [31].

For sensitivity analysis, after deleting the subset of the Micro/ITC and Macro groups, comparing the SLNB group with the ALND group, the HR of OS was 1.01 (95% CI: 0.92- 1.10, *p* = 0.85). In addition, according to the funnel plot analysis (Fig. 5B), no publication bias was found in the included studies for this outcome.

### Adverse events

Lymphedema was reported in three papers [13, 30, 53]. The OR of lymphedema rate that compared the SLNB group with the ALND group was 0.35 (95% CI: 0.25–0.49) with *p* < 0.001. The pooled results from two papers for sensory neuropathy showed that the OR of sensory neuropathy rate that compared the SLNB group with the ALND group was 0.55 (95% CI: 0.39–0.79) with *p* < 0.001 [13, 41]. Lucci et al. reported the adjusted OR of wound infections, axillary seromas, and axillary paresthesia that compared the SLNB group with the ALND group was 0.33 (95% CI: 0.16–0.68), 0.37 (95% CI: 0.22–0.63), and 0.15 (95% CI: 0.10–0.22), respectively [30]. Based on the data reported by Galimberti et al., the OR of motor neuropathy that compared the SLNB group with the ALND group was 0.33 (95% CI: 0.17–0.62) [13].

## Discussion

Based on the meta-analysis of the present review, the risk of axillary recurrence, disease progress, and overall mortality did not increase when patients with clinically node-negative but SLN-positive breast cancer were treated with SLNB only versus SLNB plus ALND. However, the SLNB group shew a benefit in locoregional recurrence than the ALND group does. In addition, the Macro subset of patients showed increased overall mortality (HR = 0.91, 95% CI: 0.83–1.00, *p* = 0.04). Compared with SLNB only, ALND was associated with an increased risk of adverse events (e.g., lymphoedema, sensory neuropathy, wound infections, axillary seromas, axillary paresthesia, and motor neuropathy) with a statistical significance.

In a meta-analysis published in 2015, Joyce et al. confirmed a significant benefit of ALND in the local control of axillary disease (OR = 2.25, 95% CI: 1.28–3.94, *p* = 0.0047) and OS (OR = 1.22, 95% CI: 1.03–1.44, *p* = 0.02) for invasive breast cancer patients [10]. However, the patients included in this meta-analysis were patients undergoing surgery for invasive breast cancer, which did not restrict patients to early-stage disease. According to our analysis, omitting ALND in patients with clinically node-negative but SLN-positive breast cancer had a similar axillary recurrence rate, DFS, and OS, but caused significantly lower incidence of adverse events compared with ALND and showed a benefit for locoregional recurrence. Our findings agreed with Chen et al. [18], although the participants and intervention criteria differed slightly. Therefore, for patients with clinically node-negative breast cancer, with SLN-positive results, omitting ALND is feasible.

Of note, during the subgroup analysis according to the histopathological classification of SLN metastasis, although there was no statistical significance, the mean OR of axillary recurrence (1.21 versus 0.91) between the two interventions was higher in the Micro/ITC subset than the overall group. Similar as the locoregional recurrence (1.02 versus 0.48). Therefore, for the Micro/ITC subset, there was a trend of axillary recurrence and locoregional recurrence with SLNB alone compared with ALND. The cause of this remains unknown, and future large-scale studies to further explore this are required.

Of note, one paper reported a significant increase in OS comparing the SLNB only group with the ALND group in patients in the Macro subset (HR = 0.91, 95% CI: 0.83–1.00, *p* = 0.04) [31]. This result indicated that for patients in the Macro subset, SLNB only had benefits for OS. However, this requires confirmation.

The advantages of this systematic review include the rigorous methodology followed in study screening, data analysis, and quality assessment. However, this study has some limitations. First, the recurrence and survival of breast cancer were largely dependent on the systematic treatments that the patients received. This was not included in our analysis and will be the focus of future research. Second, there was insufficient data to perform subgroup analysis according to the histopathological classification of SLN metastasis. In particular, the lack of evidence in the Macro subset. Next, not all the studies supply the information of the number of the positive SLN and the neoadjuvant therapy. Though our review selected studies that compared SNLB + ALND to SNLB, we were unable to control other circumstances surrounding the patient’s treatment, such as other surgical techniques and neoadjuvant therapy. Finally, only three studies were RCTs, and most of the included studies were retrospective cohort studies. Therefore, the quality of the evidence could reduce the impact of the findings from this review.

This review confirmed the evidence base for the feasibility of omitting ALND for patients with clinically node-negative but SLN-positive breast cancer. Evidence shows that although the risk of axillary recurrence, disease progress, and overall mortality was not increased when those patients were treated with SLNB alone versus SLNB and ALND, there were benefits of less adverse events and low locoregional recurrence. An OS benefit was found in the Macro subset using SLNB alone versus SLNB and ALND. Future research should focus on exploring the independent predictors for the interventions.

## Conclusion

For patients with clinically node-negative but SLN-positive breast cancer (no matter the number of the positive SLN), this review showed that SLNB alone had a similar axillary recurrence rate, DFS, and OS, but caused a significantly lower incidence of adverse events and showed a benefit for the locoregional recurrence compared with ALND. An OS benefit was found in the Macro subset that used SLNB alone versus complete ALND. Therefore, omitting ALND is feasible in this setting.

## Supplementary Information

**Additional file 1.**

## Data Availability

The datasets generated and analyzed during the present study are available from the corresponding author on reasonable request.

## Abbreviations

ALND: Axillary lymph node dissection
SLN: Sentinel lymph node
SLNB: Sentinel lymph node biopsy
DFS: Disease-free survival
OS: Overall survival
OR: Odds ratio
HR: Hazard ratio
CI: Confidence interval
Macro: Macrometastasis
Micro: Micrometastasis
ITCs: Isolated tumor cells
RoB2: Risk of bias tool for randomized trials
ROBIN-I: Risk of bias in nonrandomized studies of interventions
RCT: Randomized controlled trial
