# Supplementary material for: Efficacy and safety comparison between axillary lymph node dissection with no axillary surgery in patients with sentinel node-positive breast cancer: a systematic review and meta-analysis
Source: BMC Surg. 2023 Jul 26;23:209. doi: 10.1186/s12893-023-02101-8 (PMC10369839; doi:10.1186/s12893-023-02101-8)
Supplement: Supplementary file 1 — Additional file 1. [file 12893_2023_2101_MOESM1_ESM.pdf]

Cochrane Library CENTRAL search strategy

earch Name:
Date Run: 25/02/2023 21:58:30
Comment:

ID Search Hits
#1 MeSH descriptor: [Sentinel Lymph Node Biopsy] explode all trees 432
#2 MeSH descriptor: [Breast Neoplasms] explode all trees 17473
#3 MeSH descriptor: [Lymph Node Excision] explode all trees 1904
#4 ((Axilla*)):ti,ab,kw 6344
#5 ((Lymph Node Excision)):ti,ab,kw 1971
#6 (("breast cancer")):ti,ab,kw 39240
#7 ((Sentinel Lymph Node Biopsy)):ti,ab,kw 1175
#8 breast near neoplasm* OR breast near cancer* OR breast near carcinoma* OR breast near tumor* OR breast near tumour* 44111
#9 sentinel lymph node biopsy or SLNB or SNB or SLN or (sentinel near node) 2006
#10 lymphadenectomy OR (block or lymph node or axillary) near dissection OR (block or lymph node or axillary) near clearance 60074
#11 #1 or #7 or #9 2006
#12 #2 or #6 or # 8 601430
#13 #3 or #4 or #5 or #10 63048
#14 #11 and #12 and #13 1306

Database: Ovid MEDLINE(R) ALL <1946 to February 24, 2023>
Search Strategy:

----

1 exp Axilla/ (14461)
2 Axilla*.mp. (49167)
3 1 or 2 (49167)
4 exp Breast Neoplasms/ (337030)
5 ((breast adj neoplasm*) or (breast adj cancer*) or (breast adj carcinoma*) or (breast adj tumor*) or (breast adj tumour*)).mp. (442773)
6 4 or 5 (443371)
7 exp Sentinel Lymph Node Biopsy/ (13050)
8 (sentinel lymph node biopsy or SLNB or SNB or SLN or (sentinel adj node)).mp. (21811)
9 7 or 8 (21811)
10 exp Lymph Node Excision/ (54034)
11 (lymphadenectomy or ((block or lymph node or axillary) adj dissection) or ((block or lymph node or axillary) adj clearance)).mp. (42318)
12 10 or 11 (73677)
13 3 and 6 and 9 and 12 (5535)
14 limit 13 to english language (5006)

****

Database: Embase Classic+Embase <1947 to 2023 February 24>
Search Strategy:

----

1 exp Axilla/ (13973)
2 Axilla*.mp. (84281)

3 1 or 2 (84281)
4 exp Breast Neoplasms/ (655759)
5 ((breast adj neoplasm*) or (breast adj cancer*) or (breast adj carcinoma*) or (breast adj tumor*) or (breast adj tumour*)).mp. (723207)
6 4 or 5 (734688)
7 exp Sentinel Lymph Node Biopsy/ (20805)
8 (sentinel lymph node biopsy or SLNB or SNB or SLN or (sentinel adj node)).mp. (36537)
9 7 or 8 (36537)
10 exp Lymph Node Excision/ (80112)
11 (lymphadenectomy or ((block or lymph node or axillary) adj dissection) or ((block or lymph node or axillary) adj clearance)).mp. (112448)
12 10 or 11 (112448)
13 3 and 6 and 9 and 12 (7511)
14 limit 13 to english language (6961)

****
